# A Triplex PCR Method for Distinguishing the Wild-Type African Swine Fever Virus From the Deletion Strains by Detecting the Gene Insertion

**DOI:** 10.3389/fvets.2022.921907

**Published:** 2022-06-28

**Authors:** Zhao Huang, Zhiying Xu, Haoxuan Cao, Fanliang Zeng, Heng Wang, Lang Gong, Shengxun Zhang, Sen Cao, Guihong Zhang, Zezhong Zheng

**Affiliations:** ^1^Key Laboratory of Zoonosis Prevention and Control of Guangdong Province, College of Veterinary Medicine, South China Agricultural University, Guangzhou, China; ^2^African Swine Fever Regional Laboratory of China, Guangzhou, China; ^3^Research Center for African Swine Fever Prevention and Control, South China Agricultural University, Guangzhou, China; ^4^Daguang Food Group Co., Ltd., Jiangmen, China; ^5^Haifeng Animal Disease Prevention and Control Center, Shanwei, China; ^6^Maoming Branch, Guangdong Laboratory for Lingnan Modern Agriculture, Guangdong, China; ^7^Key Laboratory of Animal Vaccine Development, Ministry of Agriculture and Rural Affairs, Guangzhou, China

**Keywords:** African swine fever virus, triplex RT-PCR, ASFV gene deletion strain, differential diagnosis, B646L gene

## Abstract

To date, there is no effective vaccine or antiviral therapy available to prevent or treat African swine fever virus (ASFV) infections. ASFV gene deletion strains have been proposed as promising anti-ASFV vaccine candidates. In recent years, most ASFV gene deletion strains worldwide have been recombinant strains expressing *EGFP* or *mCherry* as markers. Therefore, in this study, a new triplex real-time PCR (RT-PCR) method was established for the broad and accurate differentiation of ASFV wild-type *vs*. gene deletion strains. We designed three pairs of primers and probes to target *B646L, EGFP*, and *mCherry*, and RT-PCR was used to detect these three genes simultaneously. The detection method prevented non-specific amplification of porcine reproductive and respiratory syndrome virus, porcine epidemic diarrhea virus, circovirus type 2, pseudorabies virus, and classical swine fever virus genes. The minimum copy number of standard plasmid DNA detected using triplex RT-PCR was 9.49, 15.60, and 9.60 copies for *B646L, EGFP*, and *mCherry*, respectively. Importantly, of the 1646 samples analyzed in this study, 67 were positive for ASFV, all corresponding to the wild-type virus. Overall, our data show that the triplex RT-PCR method established in this study can specifically identify both ASFV wild-type and gene deletion strains.

## Introduction

African swine fever (ASF) is an acute and severe infectious disease caused by the African swine fever virus (ASFV), with a mortality rate of 100%. At present, there is no approved safe and effective vaccine, making ASF prevention and control extremely serious and challenging (1). According to the sequence variation of the C-terminal region of the capsid protein p72 encoded by *B646L*, ASFV can be classified into 22 genotypes (2). ASF was first reported in China in August 2018, caused by the ASFV genotype II strain (3).

When ASF emerges in a new area or pig population, it is usually accompanied by high mortality and rapid spread among pigs, leading to rapid outbreaks (4). Therefore, viral detection is very important for the rapid implementation of control measures. The viral detection methods available today include virus isolation, PCR detection of the viral genome, enzyme-linked immunosorbent assays (ELISAs), direct immunofluorescence tests (DIF), and the detection of viral antigens (5). Notably, virus isolation is regarded as the gold standard for ASFV detection by the Office International Des Epizooties (OIE) (6); however, this method requires multiple materials, consumes time, and is unsuitable for large-scale detection. On the contrary, PCR detection of ASFV has high efficiency, high sensitivity, and high specificity; therefore, it is widely used, especially in the context of tissues that are not suitable for virus isolation (7, 8).

At present, safe and effective anti-ASFV vaccine is unavailable in the market. However, attenuated vaccines (after ASFV gene deletion) have emerged as the most promising anti-ASFV vaccine candidates (2). For instance, Borca et al. generated the recombinant strain ASFVΔI177L by replacing *I177L* with *mCherry*, and showed that its use protected 100% of the pigs infected with the parental strain ASFV-G; of note, these authors further developed a cell line challenged with ASFVΔI177L for high-volume production (9, 10). Additionally, Chen et al. also used *mCherry* to replace *MGF505-1R-MGF505-3R*, as well as *EGFP*, to replace *CD2v* generating the strain ASFVΔCD2vΔMGF; this new attenuated strain could also protect 100% of pigs infected with the parental strain (11).

The purification of ASFV, particularly genotype II strains, is challenging since plaque formation is difficult in the context of porcine alveolar macrophages. Borca et al. have shown that fluorescent labeling is a suitable tool for the construction of ASFV gene-deficient strains for pathogenesis studies in pigs, studies of virus-macrophage interactions, and large-scale screens that require sensitive high-throughput output (12). Since, *EGFP, mCherry*, and *RFP* are commonly used as markers in ASFV gene-deficient strains, we aimed to establish a triplex RT-PCR method for the simultaneous detection of *B646L, EGFP*, and *mCherry*, and the consequent differentiation of ASFV wild-type and gene deletion strains. Such a tool might be essential in the future if ASFV gene deletion strains are established as successful anti-ASFV vaccines to prevent the mortality associated with ASF; assays that can broadly differentiate between wild-type ASFV and deletion strains will become very important.

## Materials and Methods

### Clinical Samples

Samples collected in South China from December 2018 to May 2020 were tested via multiplex PCR at the national ASF reference laboratory; 1646 clinical samples were collected, including 706 blood samples, 336 environmental swabs, 524 oral swabs, 35 tonsil samples, and 45 lymph node samples.

### Viral Nucleic Acid Samples

The nucleic acids of ASFV, *CD2v-* and *MGF-*deficient ASFV, (ASFVΔCD2vΔMGF), porcine reproductive and respiratory syndrome virus (PRRSV), classical swine fever virus (CSFV), circovirus type 2 (PCV-2), pseudorabies virus (PRV), porcine epidemic diarrhea virus (PEDV) and porcine alveolar macrophages (PAMs) were preserved in the Infectious Diseases Department of the Veterinary College of South China Agricultural University.

### Primer and Probe Design

The primers for amplifying *B646L, EGFP*, and *mCherry* were designed according to the conserved regions of the respective genes published in GenBank. Notably, the primers for amplifying *B646L* were designed according to the complete ASFV type I-XXIV sequence. The 5' end modifying group chosen for the *B646L* probe was FAM and the 3' end quenching group used was MGB. The 5' end modifying group chosen for the *EGFP* probe was HEX and the 3' end quenching group used was BHQ1. Additionally, the 5' end modifying group chosen for the *mCherry* probe was Cy5 and the 3' end quenching group used was BHQ3. Table 1 shows the sequences of the designed synthetic primer probes and the expected size of the amplicons.

**Table 1:** List of primers used in this study.

**Target**	**Sequence/Probe (5^**′**^-3^**′**^)**	**Amplicon size (bp)**	**Orientation**
OIE-B646L-F	CTGCTCATGGTATCAATCTTATCGA	250	Forward
OIE-B646L-R	GATACCACAAGATCRGCCGT		Reverse
OIE-B646L-Probe	FAM-CCACGGGAGGAATACCAACCCAGTG-TAMRA		Forward
B646L-F	CAAAGTTCTGCAGCTCTTACATACC	134	Forward
B646L-R	GTTAATATGACCACTGGGTTGGTA		Reverse
B646L-Probe	FAM-GCTTTGAAGCCACGGGAG-MGB		Forward
EGFP-F	CTACGGCAAGCTGACCCTGAAGTTCATCTG	204	Forward
EGFP-R	TAGTTGCCGTCGTCCTTGAAGAAGATGGTG		Reverse
EGFP-Probe	HEX-TTCAAGTCCGCCATGCCCGAAGGCT-BHQ1		Forward
mCherry-F	ATGCAGAAGAAGACCATGGGCTGGGAG	246	Forward
mCherry-R	GGCGCGTTCGTACTGTTCCACGATGGTGTA		Reverse
mCherry-Probe	Cy5-ACAAGGCCAAGAAGCCCGTGCAGCT-BHQ3		Forward
PRRSV-F	TTGCTAGGCCGCAAGTAC		Forward
PRRSV-R	ACGCCGGACGACAAATGC		Reverse
PRRSV-Probe	FAM-CTGGCCCCTGCCCACCAC-BHQ1		Forward
PEDV-F	GAATTCCCAAGGGCGAAAAT		Forward
PEDV-R	TTTTCGACAAATTCCGCATCT		Reverse
PEDV-Peobe	FAM-CGTAGCAGGCTTGCTTCGGACCCA- BHQ3		Forward
PRV-F	ACGCTCGGCTTCCTCTCC		Forward
PRV-R	GGTAGTCGTCGCTCTCGTG		Reverse
PRV-Peobe	FAM-TCGCGCATCGTCTGGTGCAT-BHQ1		Forward
PCV2-F	GGAGTCTGGTGACCGTTGC		Forward
PCV2-R	CCAATCACGCTTCTGCATTTT		Reverse
PCV2-Peobe	FAM-CCGCTCACTTTCAAAAGTTCAGCCA-BHQ3		Forward
CSFV-F	CCTGAGTACAGGACAGTCGTCAGT		Forward
CSFV-R	CCCTCGTCCACATAGCATCTC		Reverse
CSFV-Peobe	FAM-TTCGACGTGAGCAGAAGCCCACC-BHQ1		Forward

### DNA Extraction and Amplification

The tissue samples were ground using a grinding rod and soaked in phosphate buffer solution; the oral and nasal swabs were directly soaked in PBS. The samples were then centrifuged and the supernatants were collected to extract nucleic acids. The nucleic acids were extracted using a nucleic acid kit (Axygen, Hangzhou, China) and then reversely transcribed using a reverse transcription kit (Takara Beijing, China). All nucleic acid samples were stored at −20°C until further use.

### Triplex qPCR

Triplex RT-PCR was performed using Bio-Rad CFX Manager 3.1 (Bio-Rad, Shanghai, China). The reaction mixture was composed of 2 μL of viral DNA, 10 μL of 2 × AceQ Universal U^+^ Probe Master Mix V2 (Vazyme, China), 0.4 μL of forward and reverse primers for *B646L, EGFP*, and *mCherry* (10 μM), 0.2 μL of *B646L, EGFP*, and *mCherry* TaqMan probe (10 μM), and an appropriate volume of ddH_2_O to a final volume of 20 μL. The reaction conditions were 40 cycles at 37°C for 2 min, 95°C for 5 min, 95°C for 10 s, and 60°C for 30 s. Importantly, negative controls were used for each test.

### Standard Plasmids

The recombinant plasmids pUC57-*EGFP*, pMD18-*B646L*, and pUC57-*mCherry* were obtained from the Department of Infectious Diseases, School of Veterinary Medicine, South China Agricultural University.

### Specificity of the Triplex qPCR

The nucleic acids extracted from wild-type ASFV, ASFVΔCD2vΔMGF, PRRSV, PEDV, PCV-2, PRV, CSFV and PAMs (Negative control, NC), were assayed using the triplex RT-PCR.

### Sensitivity Verification

The concentration of the pUC57-*EGFP*, pMD18-*B646L*, and pUC57-*mCherry* standard plasmids was determined using the Thermo Nanodrop Lite apparatus (Thermofisher Scientific, Shanghai, China), and then serial dilutions (10 folds) were performed. The diluted plasmids were then subjected to triplex RT-PCR. The copy numbers of pUC57-*EGFP*, pMD18-*B646L*, and pUC57-*mCherry* were determined according to the DNA copy number calculation formula [dsDNA copy number (copies/mL) = 6.02 × 10^23^(copies/mol) × concentration (g/mL) / DNA length × 660], which were 1.17 × 10^11^ copies/μL, 1.60 × 10^11^ copies/μL, and 1.00 × 10^11^ copies/μL, respectively.

## Results

### Sequence Alignment of the Primers and Probes

**Figure 1:**
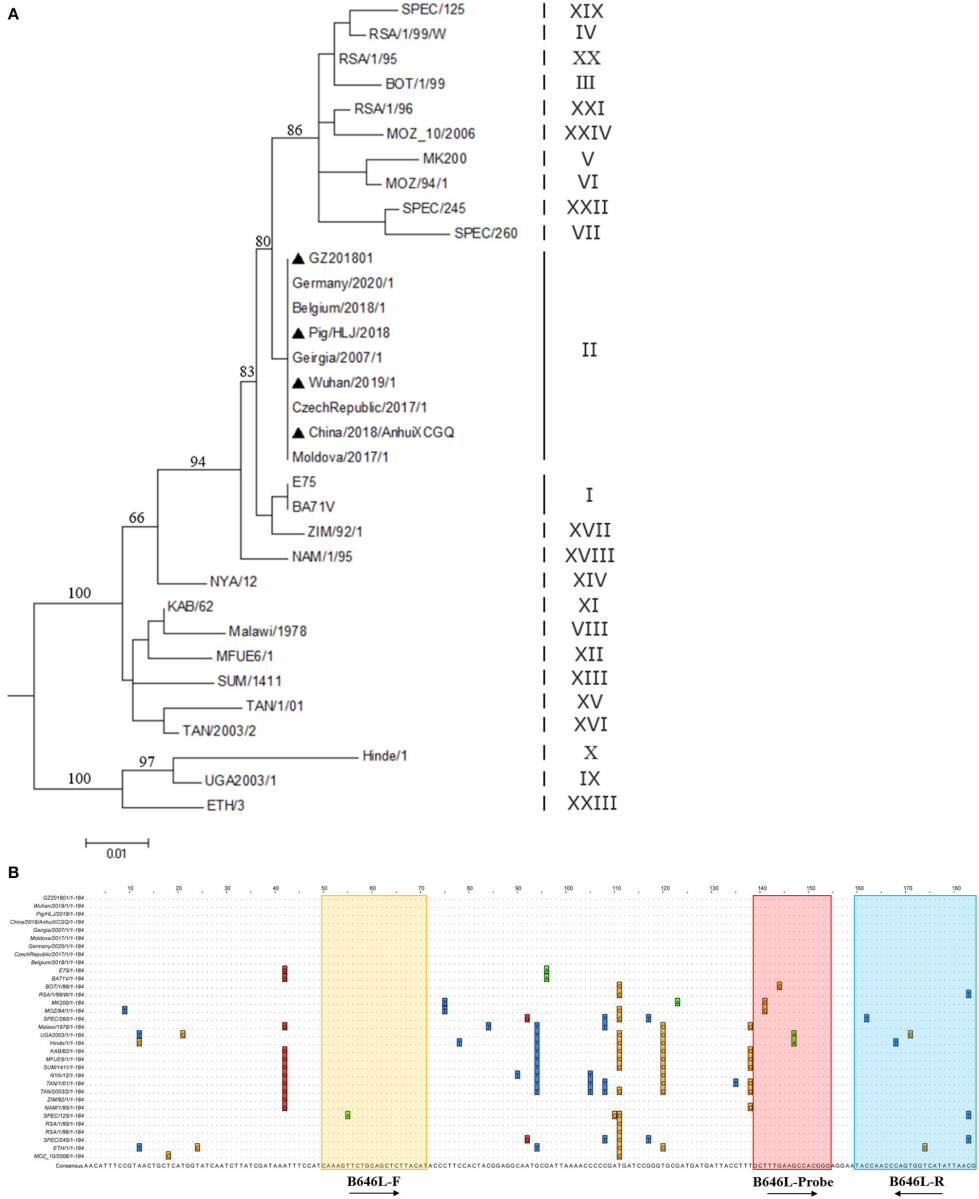
Genetic evolution of different ASFV genotypes and comparison of *B646L* sequences. **(A)** According to the B646L sequence of the 24 ASFV genotypes, the genetic evolution tree was constructed using the MEGA7.0 software. The strains marked by ▴ are the popular strains in China. **(B)** The *B646L* sequences in 24 ASFV genotypes were analyzed via Clustal W using the Megalig software (DNASTAR, Inc.).

In this study, primers and probes were designed based on the *B646L* sequence of the 24 ASFV genotypes (Figure 1); notably, the *B464L* sequence-based genetic evolution was also investigated in the context of the 24 ASFV genotypes (Figure 1A). The black triangles highlight the strains prevalent in China. Importantly, the designed probes were relatively conserved considering the *B646L* sequence of the 24 genotypes (Figure 1B), while the upstream primers showed mismatches in the the ASFV SPEC/125 genotype (Genotype XIX). Mismatches in the probes were present, particularly in the ASFV MK200 (Genotype V), MOZ/94/1 (Genotype VI), BOT/1/199 (Genotype III), Hinde1/1 (Genotype X), and UGA2003/1 (Genotype IX) genotypes. Furthermore, the downstream primers also showed mismatches in SPEC/260 (Genotype VII), UGA2003/1 (Genotype IX), Hinde/1 (Genotype X), RSA/1/99/W (Genotype IV), SPEC/125 genotype (Genotype XIX), and ETH/1 (Genotype XXIII) genotypes. Remarkably, with respect to the primers/probes designed in this study, only the SPEC/125 genotype (Genotype XIX), Hinde/1 (Genotype X) and UGA/2003/1 (Genotype IX) genotypes showed two mutations; the rest of the strains only showed one base mutation. Importantly, considering the popular Gene II strains in China, the probes/primers designed in this study did not contain any mutation sites.

### Sensitivity of the Triplex RT-PCR and Establishment of a Standard Curve

**Figure 2:**
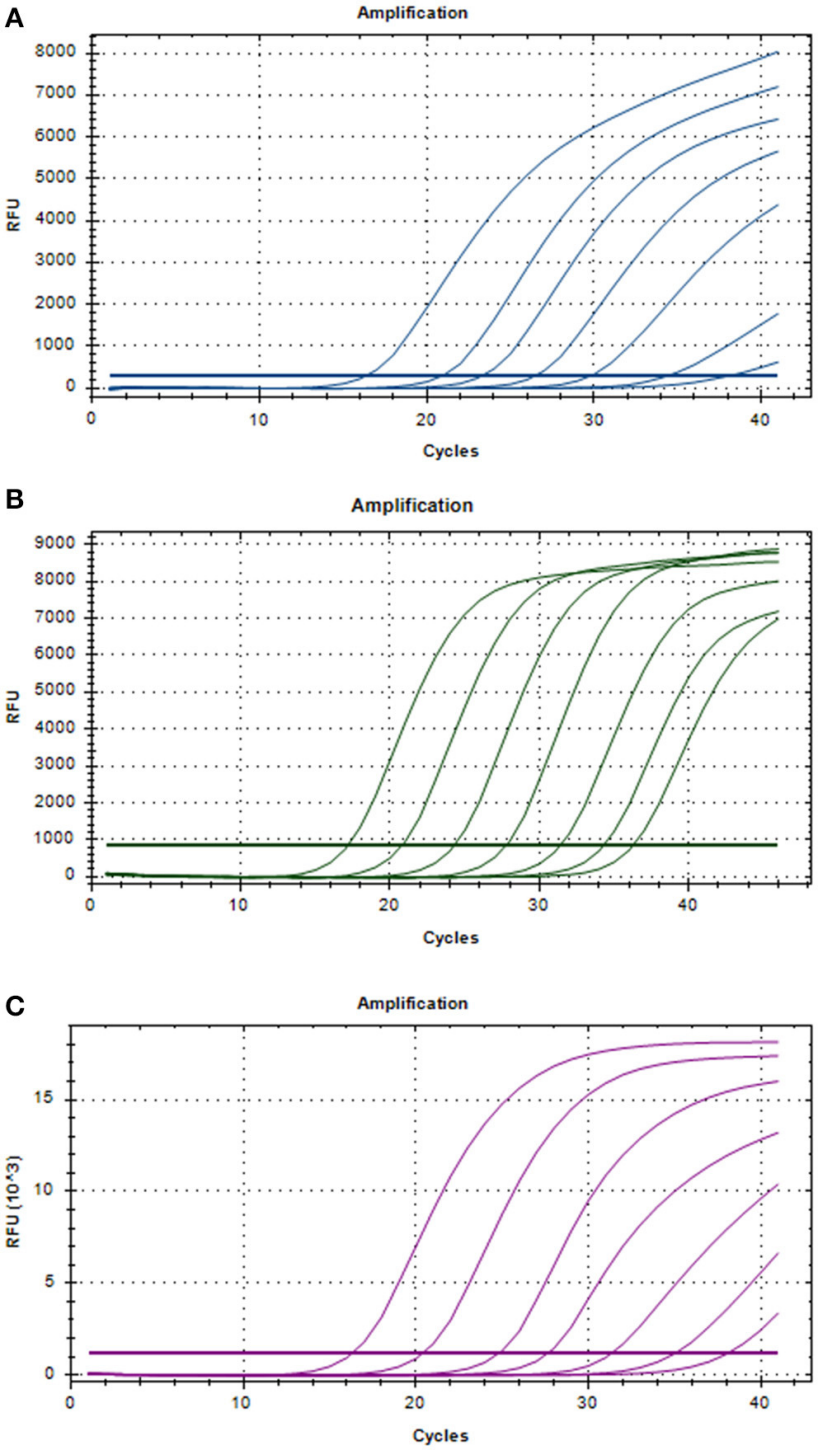
Amplification curves in the sensitivity tests of triplex RT-PCR. **(A)** Sensitivity test for B646L gene. **(B)** Sensitivity test for EGFP gene. **(C)** Sensitivity test for mCherry gene.

**Figure 3:**
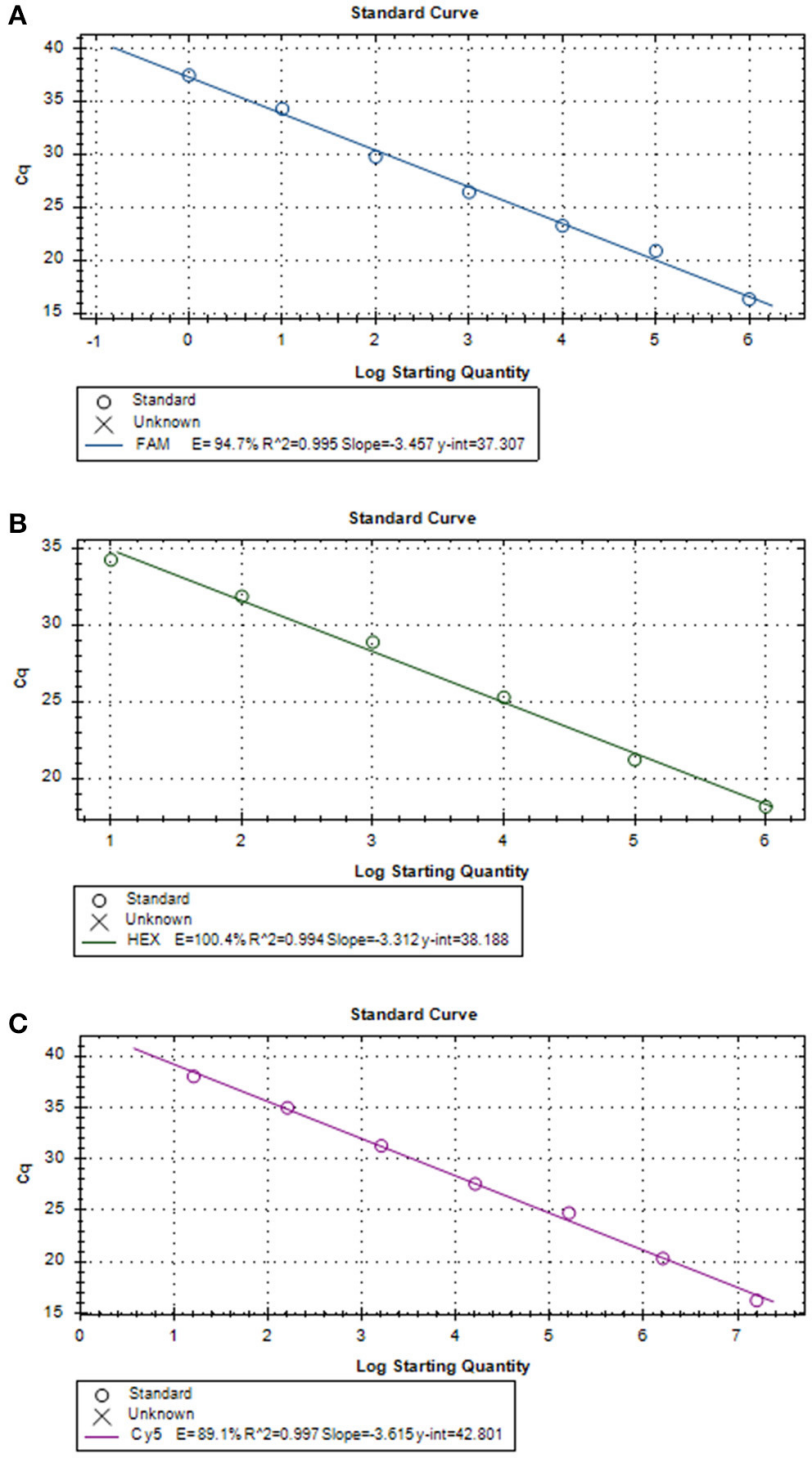
**(A)** *B646L* standard curve (y = −3.457x + 37.307; *R*^2^ = 0.995). **(B)** *EGFP* standard curve (y = −3.312x + 38.188; *R*^2^ = 0.994). **(C)** *mCherry* standard curve (y = −3.615x + 42.801; *R*^2^ = 0.997).

Three positive standard samples were serially diluted 12 times for triplex RT-PCR. Amplification was confirmed by diluting pUC57-*EGFP*, pMD18-*B646L*, and pUC57-*mCherry* plasmids (10^−4^-10^−12^), thereby obtaining the triplex RT-PCR standard curves. As shown in Figure 2, the slopes of pUC57-*EGFP*, pMD18-*B646L*, and pUC57-*mCherry* standard curves were −3.457, −3.312, −3.615, respectively. The standard curves (*R*^2^ > 0.99) showed that each diluted sample had a strong linear relationship. Importantly, the highest dilution (10^−11^) of the positive plasmid was detected using this method. Additionally, as shown in Figure 3, the minimum copies of pUC57-*EGFP*, pMD18-*B646L*, and pUC57-*mCherry* detected using triplex RT-PCR were 9.49, 15.60 and 9.60, respectively, suggesting that this method had a high detection sensitivity.

### Specificity of the Triplex Real-Time PCR

**Figure 4:**
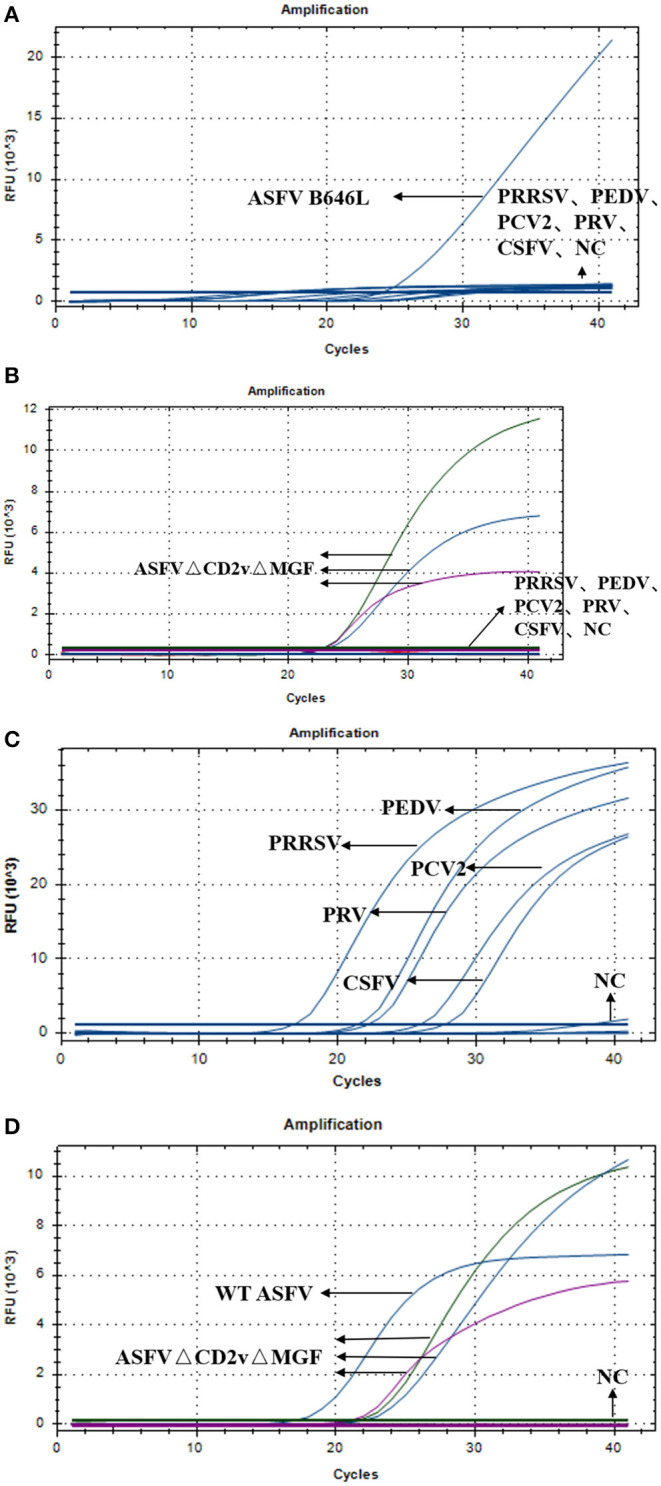
**(A)** Amplification curves of ASFV, PRRSV, PEDV, PCV-2, PRV, CSFV, and NC. **(B)** Amplification curves of ASFVΔCD2vΔMGF, PRRSV, PEDV, PCV-2, PRV, CSFV, and NC. **(C)** Amplification curve of PRRSV, PEDV, PCV-2, PRV, CSFV, and NC. **(D)** Amplification curves of ASFV, ASFVΔCD2vΔMGF and NC.

To test the specificity of the triplex real-time PCR, we used triplex RT-PCR to detect the nucleic acids of wild-type ASFV, ASFVΔCD2vΔMGF, PRRSV, PEDV, PCV-2, PRV, CSFV, and NC. As shown in Figure 4A, detection of WT-ASFV PRRSV, PEDV, PCV-2, PRV, CSFV using triplex RT-PCR, a curve was obtained for ASFV nucleic acid samples (FAM signal), while no amplification curve was obtained for PRRSV, PEDV, PCV-2, PRV, CSFV, and NC nucleic acid. As shown in Figure 4B, detection of ASFVΔCD2vΔMFG PRRSV, PEDV, PCV-2, PRV, CSFV using triplex RT-PCR, in the ASFVΔCD2vΔMFG nucleic acid samples, the FAM, HEX and Cy5 channels all showed amplification curves, corresponding to B646L, EGFP, and mCherry, respectively. Indicated that the *EGFP* and *mCherry* were expressed in ASFVΔCD2vΔMGF, which could be identified as a gene deletion virus. As shown in the Figure 4C, specific probe primers were used to detect the nucleic acids of five swine viruses, the nucleic acid samples of the five swine viruses all showed specificity curves, indicating that the nucleic acids of the five swine viruses did exist in the specifically detected samples. As shown in Figure 4D, Using triplex RT-PCR to detect WT-ASFV and ASFVΔCD2vΔMFG simultaneously, WT-ASFV showed amplification curve only in the FAM channel, while ASFVΔCD2vΔMFG showed amplification curve in FAM, HEX and Cy5 channels. The above results suggested that the established triplex real-time PCR has a good specificity without cross-reactivity to other five swine viruses.

### Triplex RT-PCR of Clinical Swine Nucleic Acid Samples

A total of 1,646 clinical swine samples were subjected to triplex RT-PCR. The results showed that 67 samples were positive for ASFV, while the remaining 1,579 samples were negative. Notably, the results for all of the 67 positive samples showed that an amplification curve could only be obtained for the FAM channel, indicating that these positive samples were ASFV wild-type strains. This result was expected since the attenuated strains are not yet used as vaccines. Importantly, the same results were obtained using the primers and probes published by OIE (Table 2), validating the triplex RT-PCR designed in this study as a sensitive and specific tool for the identification of ASFV.

**Table 2:** Identification of the 67 ASFV clinical positive samples was performed using the ASFV real-time PCR method.

**No**.	**Sample type**	**Province of sampling**	**Date of sampling**	**OIE recommend real-time PCR (Ct value)**	**Triplex real-time PCR in this study (Ct value)**		
					***B646L* Gene**	***EGFP* Gene**	***mCherry* Gene**
1	Blood	Guangdong	2018/12/23	15.36	15.1	-	-
2	Blood	Guangdong	2018/12/23	16.9	16.8	-	-
3	Blood	Guangdong	2018/12/23	20.0	19.9	-	-
4	Lymph nodes	Guangdong	2018/12/23	23.62	23.45	-	-
5	Lymph nodes	Guangdong	2018/12/23	23.45	23.25	-	-
6	Blood	Guangdong	2018/12/25	27.53	27.5	-	-
7	Blood	Guangdong	2018/12/25	23.61	23.56	-	-
8	Lymph nodes	Guangdong	2018/12/25	26.42	26.7	-	-
9	Lymph nodes	Guangdong	2018/12/25	26.43	26.45	-	-
10	Oral swabs	Guizhou	2019/6/23	35.6	35.4	-	-
11	Oral swabs	Guizhou	2019/6/23	33.6	33.45	-	-
12	Oral swabs	Guizhou	2019/6/23	33.42	33.24	-	-
13	Blood	Guizhou	2019/6/23	30.25	31.1	-	-
14	Blood	Guizhou	2019/6/23	25.32	25.26	-	-
15	Blood	Guizhou	2019/6/23	29.28	29.76	-	-
16	Blood	Guizhou	2019/6/23	26.35	26.2	-	-
17	Blood	Guizhou	2019/6/23	21.36	21.98	-	-
18	Oral swabs	Yunnan	2019/4/4	30.29	30.32	-	-
19	Oral swabs	Yunnan	2019/4/4	34.26	35.02	-	-
20	Blood	Yunnan	2019/4/4	26.31	26.41	-	-
21	Blood	Yunnan	2019/4/4	25.72	25.76	-	-
22	Blood	Yunnan	2019/5/25	30.2	30.28	-	-
23	Blood	Yunnan	2019/5/25	30.25	20.26	-	-
24	Blood	Yunnan	2019/5/25	26.7	26.76	-	-
25	Blood	Yunnan	2019/5/25	26.96	27.1	-	-
26	Blood	Yunnan	2019/5/25	24.3	24.42	-	-
27	Blood	Yunnan	2019/5/25	25.67	25.7	-	-
28	Blood	Yunnan	2019/5/25	26.16	26.32	-	-
29	Oral swabs	Yunnan	2019/10/25	35.64	35.26	-	-
30	Oral swabs	Yunnan	2019/10/25	37.65	36.98	-	-
31	Oral swabs	Yunnan	2019/10/25	35.24	35.39	-	-
32	Oral swabs	Yunnan	2019/10/25	32.06	32.2	-	-
33	Oral swabs	Yunnan	2019/10/25	28.67	28.68	-	-
34	Blood	Guangxi	2019/2/19	19.89	20.1	-	-
35	Blood	Guangxi	2019/2/19	20.56	20.73	-	-
36	Blood	Guangxi	2019/2/19	18.65	18.42	-	-
37	Blood	Guangxi	2019/2/19	23.24	23.36	-	-
38	Blood	Guangxi	2019/5/27	20.69	20.56	-	-
39	Blood	Guangxi	2019/5/27	21.68	21.98	-	-
40	Lymph nodes	Guangxi	2019/5/27	16.7	16.5	-	-
41	Lymph nodes	Guangxi	2019/5/27	17.56	17.32	-	-
42	Environmental swabs	Guangxi	2019/8/8	32.89	32.49	-	-
43	Environmental swabs	Guangxi	2019/8/8	29.86	29.87	-	-
44	Environmental swabs	Guangxi	2019/8/8	28.49	28.32	-	-
45	Environmental swabs	Guangxi	2019/8/8	30.26	30.1	-	-
46	Environmental swabs	Guangxi	2019/10/15	32.98	33.19	-	-
47	Environmental swabs	Guangxi	2019/10/15	31.59	31.26	-	-
48	Environmental swabs	Guangxi	2019/10/15	35.62	35.23	-	-
49	Environmental swabs	Guangxi	2019/10/15	29.84	29.78	-	-
50	Environmental swabs	Guangxi	2019/10/15	28.56	28.64	-	-
51	Environmental swabs	Guangxi	2019/10/15	30.48	30.98	-	-
52	Lymph nodes	Fujian	2018/12/24	25.63	25.36	-	-
53	Lymph nodes	Fujian	2018/12/24	20.56	25.5	-	-
54	Blood	Fujian	2018/12/24	25.67	26.1	-	-
55	Blood	Fujian	2018/12/24	23.19	23.35	-	-
56	Blood	Fujian	2018/12/24	22.68	22.16	-	-
57	Blood	Hainan	2019/4/19	29.56	29.46	-	-
58	Blood	Hainan	2019/4/19	32.16	32.1	-	-
59	Blood	Hainan	2019/4/19	25.68	25.79	-	-
60	Blood	Hainan	2019/4/19	26.49	26.59	-	-
61	Blood	Hainan	2019/4/21	19.56	19.46	-	-
62	Blood	Hainan	2019/4/21	24.39	24.79	-	-
63	Blood	Hainan	2019/4/21	25.79	25.76	-	-
64	Lymph nodes	Hainan	2019/4/21	22.12	22.32	-	-
65	Oral swabs	Hainan	2019/4/21	32.19	32.73	-	-
66	Oral swabs	Hainan	2019/4/21	29.36	29.56	-	-
67	Oral swabs	Hainan	2019/4/21	29.76	29.37	-	-

## Discussion

Here, a new triplex RT-PCR method was developed to detect *B646L, EGFP*, and *mCherry*, and the consequent distinction between wild-type and gene-deleted ASFV strains. If only *B646L* is detected, the sample must contain only wild-type ASFV. On the contrary, if both *B646L* and either *EGFP* or *mCherry* are detected, the sample must derive from a gene deletion virus strain. It should be noted that *B646L* must be detected in both wild-type and gene-deleted ASFV strains; otherwise, it would mean that the sample is negative ASFV nucleic acids.

In this study, we designed a pair of primers and probes able to target *B646L* of all ASFV genotypes for the first time. Notably, a one-base mismatch existed in the genotypes III, V, VI, VII, IV, XIX, and XXIII, and a two-base mismatch was observed considering genotypes IX and X. Ghedira et al. showed that mismatches between primers and templates have different effects on amplification efficiency; mismatches near the 3' end can lead to low amplification efficiency, while mismatches outside the two bases of the 3' end have little effect (13). Importantly, the mismatches observed using the primers/probes designed in this study did not include the two bases at the 3' end. Therefore, we speculated that the amplification efficiency would not be affected and consequently, the triplex RT-PCR designed in this study would amplify *B646L* of the different ASFV genotypes in circulation.

ASF is a severe infectious disease of swine that causes huge economic losses to the breeding industry. Biosafety and vaccines are the most effective and economically-viable methods for preventing and controlling ASF epidemics. Monitoring whether pigs are infected by pathogens is the most important aspect of biosafety. On the contrary, vaccines are expected to decrease the disease burden. However, to date, there are no vaccines available. Still, gene deletion attenuated ASFV strains have become the most promising anti-ASFV vaccine candidates. In the review part of this paper, we listed some ASFV gene deletion vaccines that can protect pigs from virulent strains, such as ASFVΔMGFΔCD2v and ASFVΔI177L strain. Importantly, if the ASFV gene deletion strains are successfully marketed, it is necessary to distinguish them from the wild-type ASFV accurately.

At present, a variety of fluorescence quantitative PCR methods have been developed to detect ASFV gene deletion strains (14–16). However, most of the above methods are aimed at a single deleted gene, which has great limitations. Therefore, the method developed in this study, which we used to detect a variety of gene deletion strains, can be a valuable new tool with a wider detection range.

Importantly, the specificity results showed that this new method does not lead to non-specific results for common viruses circulating in pig farms. Additionally, the lowest number of copies of the positive standard for the detection of *B646L, EGFP*, and *mCherry* were 9.49, 15.60, and 9.60, respectively, and the established standard curves showed a strong linear relationship. Therefore, overall, our experimental data strongly support the accuracy and reliability of the new triplex RT-PCR developed here.

A new triplex fluorescent quantitative PCR method was successfully established for the identification of both wild-type and gene deletion ASFV strains, as well as their distinction. Importantly, the results of this method are consistent with those obtained with the OIE-recommended method. Altogether, our results suggest that the reliable method developed in this study can be used as a technical reserve to identify the ASFV gene deletion vaccines as well as wild-type II ASFV.

## Data Availability Statement

The original contributions presented in the study are included in the article/supplementary material, further inquiries can be directed to the corresponding author/s.

## Author Contributions

ZH, ZX, HC, and FZ: conceptualization and writing—original draft preparation. ZZ, GZ, HW, and LG: data curation and visualization. ZH, ZX, HC, FZ, and SZ: validation and methodology. ZZ and GZ: funding. All authors have read and agreed to the published version of the final manuscript.

## Funding

This research was funded by the Key-Area Research and Development Program of Guangdong Province (2019B020211003), the National Key Research and Development Program of China (2021YFD1800101). Start-up Research Project of Maoming Laboratory (2021TDQD002) and China Agriculture Research System of MOF and MARA (CARS-35).

## Conflict of Interest

SZ was employed by Daguang Food Group Co., Ltd. The remaining authors declare that the research was conducted in the absence of any commercial or financial relationships that could be construed as a potential conflict of interest.

## Publisher's Note

All claims expressed in this article are solely those of the authors and do not necessarily represent those of their affiliated organizations, or those of the publisher, the editors and the reviewers. Any product that may be evaluated in this article, or claim that may be made by its manufacturer, is not guaranteed or endorsed by the publisher.
